# Functional characterization of a lytic polysaccharide monooxygenase from the thermophilic fungus *Myceliophthora thermophila*

**DOI:** 10.1371/journal.pone.0202148

**Published:** 2018-08-20

**Authors:** Marco A. S. Kadowaki, Anikó Várnai, John-Kristian Jameson, Ana E. T. Leite, Antonio J. Costa-Filho, Patricia S. Kumagai, Rolf A. Prade, Igor Polikarpov, Vincent G. H. Eijsink

**Affiliations:** 1 Department of Physics and Interdisciplinary Science, São Carlos Institute of Physics, University of São Paulo, São Carlos, São Paulo, Brazil; 2 Faculty of Chemistry, Biotechnology and Food Science, Norwegian University of Life Sciences (NMBU), Ås, Norway; 3 Department of Physics, Faculdade de Filosofia, Ciências e Letras de Ribeirão Preto, University of São Paulo, Ribeirão Preto, São Paulo, Brazil; 4 Departments of Biochemistry & Molecular Biology and Microbiology & Molecular Genetics, Oklahoma State University, Stillwater, OK, United States; Institut National de la Recherche Agronomique, FRANCE

## Abstract

Thermophilic fungi are a promising source of thermostable enzymes able to hydrolytically or oxidatively degrade plant cell wall components. Among these enzymes are lytic polysaccharide monooxygenases (LPMOs), enzymes capable of enhancing biomass hydrolysis through an oxidative mechanism. *Myceliophthora thermophila* (synonym *Sporotrichum thermophile*), an Ascomycete fungus, expresses and secretes over a dozen different LPMOs. In this study, we report the overexpression and biochemical study of a previously uncharacterized LPMO (*Mt*LPMO9J) from *M*. *thermophila* M77 in *Aspergillus nidulans*. *Mt*LPMO9J is a single-domain LPMO and has 63% sequence similarity with the catalytic domain of *Nc*LPMO9C from *Neurospora crassa*. Biochemical characterization of *Mt*LPMO9J revealed that it performs C4-oxidation and is active against cellulose, soluble cello-oligosaccharides and xyloglucan. Moreover, biophysical studies showed that *Mt*LPMO9J is structurally stable at pH above 5 and at temperatures up to 50°C. Importantly, LC-MS analysis of the peptides after tryptic digestion of the recombinantly produced protein revealed not only the correct processing of the signal peptide and methylation of the N-terminal histidine, but also partial autoxidation of the catalytic center. This shows that redox conditions need to be controlled, not only during LPMO reactions but also during protein production, to protect LPMOs from oxidative damage.

## Introduction

Lytic Polysaccharide Monooxygenases (LPMOs) are oxidative enzymes able to boost the hydrolytic efficiency of glycoside hydrolases (GHs) during the depolymerization of recalcitrant polysaccharides (such as lignocellulose and chitin) [1, 2]. LPMOs are key components of the latest generation commercial cellulase cocktails, which also include a core set of cellobiohydrolases, endoglucanases and β-glucosidases [3]. Although the contribution of additional factors [4] and redox enzymes [5] has been predicted since 1950, the existence and structure of LPMOs have been described just recently [2, 6–8], and their mechanism is still under debate [9–11]. LPMOs are metalloenzymes, with a Cu(I/II) ion coordinated by two histidines forming a His-brace in the active site [2, 6]. In fungal LPMOs, one of the coordinating histidines, the N-terminal His residue (His1), is methylated at Nε2 [6].

LPMO action requires an external electron donor [2] and molecular oxygen [2] or hydrogen peroxide [10]. Electron donors may be non-enzymatic (such as ascorbate and lignin-derived phenolic compounds [2, 12–14]) or enzymatic (e.g. cellobiose dehydrogenase [14–18]). Cleavage of β-1,4-glycosidic bonds by LPMOs involves oxidation of either the C1 or the C4 carbon in the scissile bond, whereas some LPMOs show mixed C1/C4 activity leading to the production of a mixture of C1-, C4-, and double-oxidized products [19]. The attack of LPMOs on the surface of crystalline cellulose disrupts the crystalline structure and introduces new binding sites for cellulases [20, 21].

Based on their sequences, LPMOs are currently classified within six families of Auxiliary Activity (AA) enzymes in the Carbohydrate-Active enZymes (CAZy) database (http://www.cazy.org) [22]: AA9, AA11, AA13, and AA14 found in fungi, AA15 mainly found in insects but also in viruses, and AA10 mainly found in bacteria but also in other organisms. LPMOs belonging to the AA9 family, also called LPMO9s, have shown activity on a variety of substrates, including cellulose [6], several hemicellulose β-glucans [23] and cello-oligosaccharides [24]. LPMOs are highly redundant in the genomes of ascomycetes and it is conceivable that this redundancy reflects functional diversity that is needed to degrade complex substrates such as plant cell walls [14, 25]. As an example, the genome of the filamentous fungus *Myceliophthora thermophila*, which is a relevant industrial host for production of thermostable enzymes, encodes 23 AA9 LPMOs–see Table A in S1 File for the complete list of *Mt*LPMO9s [26] as well as the CAZy database (http://www.cazy.org) and JGI’s genome portal (https://genome.jgi.doe.gov/portal/). Of these, 11 have been detected in the secretome of *M*. *thermophila* C1 grown on alfalfa and barley straw [26] and five *Mt*LPMO9s (four homologously expressed in *M*. *thermophila* C1) have been characterized to date [13, 19, 27] (see also Table A in S1 File).

In the present study, we report the functional and structural characterization of an AA9 LPMO from *M*. *thermophila* (syn. *Sporotrichum thermophile*), termed *Mt*LPMO9J. The enzyme was heterologously expressed in *Aspergillus nidulans*, purified and then characterized using a variety of methods, assessing properties such as substrate specificity, oxidative regioselectivity and stability. *Mt*LPMO9J stands out as being one of the few LPMOs demonstrating activity on soluble cello-oligosaccharides [24, 28, 29].

## Materials and methods

### Enzyme

#### Sequence alignment and phylogenetic analysis

**Fig 1:**
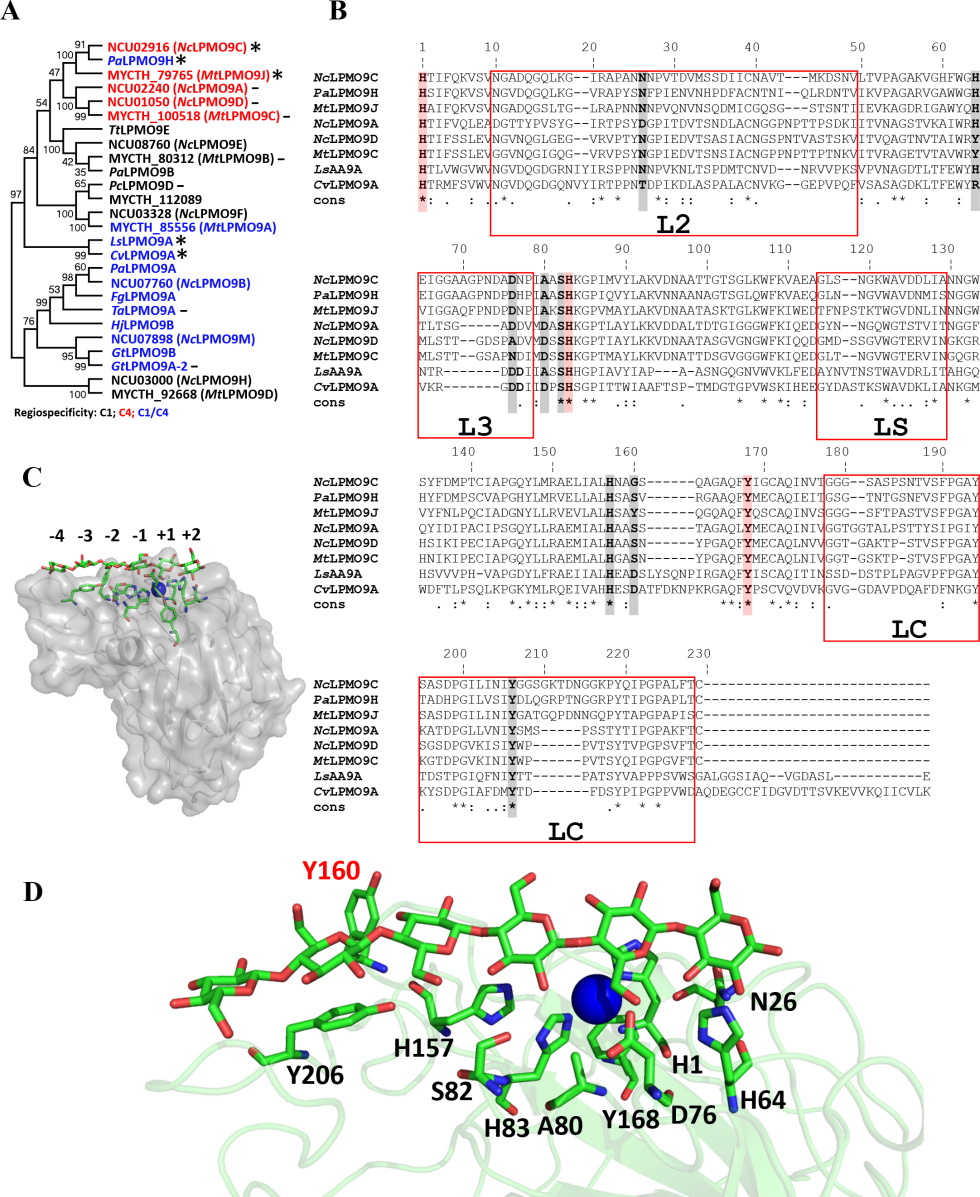
Sequence-based and structural comparison of LPMO9s. (A) Phylogenetic tree of characterized LPMO9s, based on alignment of catalytic modules only. The oxidative regiospecificity (C1, black; C4, red; C1/C4, blue) is indicated for each LPMO. LPMOs active on soluble cello-oligosaccharides are indicated by asterisk (*) and LPMOs reported to be unable to cleave cello-oligosaccharides are indicated by dash (–). (B) Structure based multiple sequence alignment (MSA) of *Mt*LPMO9J with C4-oxidizing and cello-oligosaccharide-active LPMOs. The conserved residues involved in copper ion coordination are shaded in red; residues involved in substrate binding in *Ls*LPMO9A as well as Tyr160 in *Mt*LPMO9J are shaded in grey. Red boxes indicate variable regions in the LPMO9 family, which have been given the indicated names [46]. Note that several of the residues potentially involved in substrate binding occur in the most variable LPMO regions, where the alignment is somewhat uncertain despite being largely structure based. (C) Structural model of *Mt*LPMO9J (in grey, with selected side chains in green) built with the Swiss-Model Server using the crystal structure of *Nc*LPMO9C (PDB:4D7U) as template. The cellohexaose (green carbons) was modelled by superposition of the model with the crystal structure of *Ls*AA9A from *Lentinus similis* in complex with cellohexaose (PDB:5ACI; [35]). The copper ion is shown as a blue sphere. (D) Close-up of residues potentially involved in substrate binding in *Mt*LPMO9J. Tyr160, which is unique for *Mt*LPMO9J has a red label.

*Mt*LPMO9J (MYCTH_79765, UniProt: G2Q7A5) from *M*. *thermophila* was characterized. Phylogenetic analysis of *Mt*LPMO9J was performed using sequence alignment generated with MUSCLE [30] and MEGA5 software [31] and the neighbor-joining method. The consensus tree was inferred using a bootstrap of 1000 replicates. The structure based sequence alignment shown in Fig 1 was generated with T-Coffee [32].

#### Homology model

A structural model of *Mt*LPMO9J was generated using the Swiss-Model Automated Comparative Protein Server [33] and the crystal structure of *Nc*LPMO9C from *N*. *crassa* (PDB:4D7U) [34] as a template. The cellohexaose complex model was generated by superposition with the crystal structure of *Ls*AA9A from *Lentinus similis* (PDB:5ACI; [35]). The images were generated using the PyMOL Molecular Graphics System (Version 1.5.0.4 Schrödinger, LLC, New York, NY, USA).

#### Cloning, expression and purification of *Mt*LPMO9J

The gene encoding *Mt*LPMO9J (MYCTH_79765) from *M*. *thermophila* strain M77 [36] was amplified from genomic DNA, including the native signal peptide using primers 5’-agcatcattacacctcagcaATGAAGCTCTCCCTCTTTTC-3’ (forward) and 5’-taaatcactagatatctctaTCAGCAGGAGATGGGCGCGG-3’ (reverse), and cloned into the pEXPYR vector [37] using Ligation-Independent Cloning [38]. The expression plasmid was transformed into *A*. *nidulans* A773 (*pyrG*89; *wA*3; *pyroA*4) as described earlier [37].

Approximately 10^7^ spores/mL were used to inoculate in 3 L liquid minimal medium, pH 6.5, containing 50 mL/L Clutterbuck salts (120 g/L NaNO_3_, 10.4 g/L KCl, 10.4 g/L MgSO_4_·7H_2_O and 30.4 g/L of KH_2_PO_4_), 1 mL/L trace elements (22 g/L of ZnSO_4_·7H_2_O, 11 g/L of H_3_BO_3_, 5 g/L of MnCl_2_·4H_2_O, 5 g/L of FeSO_4_·7H_2_O, 1.6 g/L of CoCl_2_·5H_2_O, 1.6 g/L of CuSO_4_·5H_2_O, 1.1 g/L of Na_2_MoO_4_·4H_2_O and 50 g/L of Na_2_EDTA), supplemented with 1 mg/L pyridoxine and 5% (w/v) maltose and maintained in static culture at 37°C for 48 h. The culture medium was filtered using Miracloth membrane (Calbiochem, San Diego, CA, USA) with a pore size of 22–25 μM, and the secreted proteins were concentrated 10-fold by tangential flow filtration using a hollow fiber cartridge with 5,000 molecular weight cutoff (GE Healthcare, Uppsala, Sweden). The concentrated protein solution (approx. 100 mL) was immediately applied to a 10 mL DEAE-Sephadex column (GE Healthcare) pre-equilibrated with 20 mM Tris/HCl buffer pH 8.0 and *Mt*LPMO9J was collected in the flow through fraction. The flow through fraction was concentrated by ultrafiltration using a centrifugal filter concentrator with 10,000 molecular weight cutoff (Millipore, Billerica, MA, USA). The NaCl concentration was adjusted to 150 mM and *Mt*LPMO9J was further purified using a HiLoad 16/60 Sephadex75 size exclusion column (GE Healthcare) equilibrated with 20 mM Tris/HCl buffer pH 8.0 containing 150 mM NaCl. Protein concentrations were determined spectrophotometrically at 280 nm using a molar extinction coefficient of 41160 M^-1^ cm^-1^ [39]. Protein purity was analyzed by SDS-PAGE [40] using Coomassie Brilliant Blue G-250 staining (Sigma, Deisenhofen, Germany), and the protein identity was confirmed by mass spectrometry (see below).

### Analysis of purified *Mt*LPMO9J

#### HPLC-MS/MS analysis

Purified *Mt*LPMO9J (10 μg in 50 μL 20 mM Tris/HCl pH 8.0) was subjected to digestion with trypsin followed by reversed phase peptide clean-up, and the peptides were analyzed according to the method described by Arntzen *et al*. [41]. The peptides were fractionated using a nanoLC system (Dionex Ultimate 3000 UHPLC; Thermo Scientific, Bremen, Germany), equipped with an Acclaim PepMap100 column (C_18_, 5 μm, 100 Å, 300 μm i.d. x 5 mm, Thermo Scientific). At the start, the column was equilibrated in a mixture of 96% solution A [0.1% (v/v) formic acid] and 4% solution B [80% (v/v) ACN, 0.08% (v/v) formic acid]. Peptides were eluted using a 40 min gradient developing from 4% to 15% (v/v) solution B in 2 min and from 15% to 55% (v/v) solution B in 25 min before a wash phase at 90% solution B, all at a flow rate of 300 nL/min. The column was connected to a Q-Exactive mass spectrometer (Thermo Fischer Scientific, Rockford, IL, USA) operated in data-dependent mode to switch automatically between orbitrap-MS and higher-energy collisional dissociation (HCD) orbitrap-MS/MS acquisition. Peptides were identified with the Mascot server (version 2.6.1) against *Mt*LPMO9J and the *Aspergillus nidulans* proteome, the latter being the background from the expression host. Correct processing of the signal peptide (i.e. cleavage before His1) was evaluated using the SEMI-trypsin search on the full-length *Mt*LPMO9J sequence, which allowed identification of fragments resulting from one non-tryptic cleavage (i.e. cleavage of the signal peptide) at the N-terminus of the peptide and one trypsin-specific cleavage at the C-terminus of the peptide (http://massqc.proteomesoftware.com/help/metrics/percent_semi_tryptic). Tryptic peptides and their possible modifications were identified using error tolerant Mascot searches on the *Mt*LPMO9J sequence without the signal peptide.

#### Electron paramagnetic resonance (EPR) spectroscopy

EPR experiments were performed on a Varian E109 spectrometer equipped with a cryogenic system, which allowed for low-temperature data collection. The spectrometer was operated at 9.26 GHz, with a modulation amplitude of 4 G and microwave power of 10 mW, at 70 K. Samples were drawn into quartz tubes and then frozen in liquid nitrogen. The EPR parameters were optimized to avoid line saturation and distortion. The spectrum of the buffer only was used as a baseline and subtracted from all other spectra.

#### Circular dichroism

Circular dichroism (CD) measurements were conducted on a JASCO J-815 spectropolarimeter (JASCO Inc., Maryland, USA) using 0.5 mg/mL of purified *Mt*LPMO9J in 20 mM Na-phosphate buffer pH 6.0. The sample was analysed at 25°C using a 0.1 cm path length quartz cell. The far-UV spectra were recorded using a wavelength range of 205–260 nm, with 50 nm/min scan speed and 1 nm band width. The final data were obtained by signal averaging six spectra before subtracting the buffer spectrum and are reported as mean residue ellipticity (MRW). The thermal denaturation experiment was performed following the ellipticity (*θ*) at wavelength 215 nm, corresponding to the minimum value of the spectra, at a constant heating rate of 1°C/min with 0.5 mg/mL *Mt*LPMO9J from 20 to 90°C.

#### ThermoFluor assay

A ThermoFluor assay was applied to study the thermal stability of *Mt*LPMO9J in a set of 8 buffers, each at a concentration of 50 mM, with the pH ranging from 2.0 to 9.0. Reactions with 62 μM *Mt*LPMO9J and 0.05% (v/v) SYPRO Orange dye (Sigma, Deisenhofen, Germany) were prepared in each buffer on a 96-well plate [42]. The plate was sealed with Optical-Quality Sealing Tape (Bio-Rad, Veenendaal, The Netherlands) and heated in an iCycler iQ Real-Time PCR Detection System (Bio-Rad) from 25 to 90°C, with stepwise increments of 1°C per min and a 30 s hold step for every point, followed by fluorescence reading with excitation/emission wavelengths at 490/530 nm.

### *Mt*LPMO9J activity measurements

#### Substrates

The following substrates were used: phosphoric acid swollen cellulose (PASC, prepared from Avicel PH-101 (Sigma, Deisenhofen, Germany), as described earlier [43]), cellotriose, cellotetraose, cellopentaose, and cellohexaose (Megazyme, Wicklow, Ireland), and the hemicelluloses sugar beet arabinan, konjac glucomannan, barley β-glucan, tamarind xyloglucan (XG) (Megazyme) and beechwood xylan (Sigma, Deisenhofen, Germany).

#### Enzyme reactions

The substrates (0.1%, w/v) were incubated with *Mt*LPMO9J (5 μM) in 20 mM sodium acetate buffer (pH 6.0) at 50°C and shaking at 1000 rpm, in 100 μl total volume for up to 16 h. In all experiments, 1 mM ascorbic acid was used as reducing agent. For the time course experiments, 100 μL aliquots were removed from reactions with 1 mL total volume at defined time intervals. Control reactions were set up without reducing agent or enzyme; reactions containing substrate only were also set up. As a positive control, *Nc*LPMO9C was used [23]. Reactions were stopped by boiling for 10 min, then the supernatants were separated from insoluble substrates by filtration using a 96-well filter plate (Millipore, MA, USA) and a vacuum manifold system (Millipore). The samples were analyzed by HPAEC-PAD and/or MALDI-TOF/MS (for details, see below).

#### Analysis of LPMO products with HPAEC-PAD

LPMO reaction products were analyzed by high-performance anion exchange chromatography (HPAEC) on a Dionex ICS3000 instrument equipped with pulsed amperometric detection (PAD) and a CarboPac PA1 column (2×250 mm) with a CarboPac PA1 guard column (2×50 mm). For analysis of cellulosic substrates, a 50-min gradient [44] and cello-oligosaccharide standards (Megazyme) were used. In the case of hemicellulosic substrates, we used a 75-min gradient [23] and purified XG7 (XXXG) and XG oligosaccharides (Megazyme) as standards. In the time course experiments, oxidized products of *Mt*LPMO9J on PASC were quantified by integrating the peak areas of C4-oxidized products.

#### MALDI-TOF/MS

LPMO reaction products were also analyzed using MALDI-TOF mass spectrometry on an Ultraflex MALDI-ToF/ToF instrument (Bruker Daltonics, Bremen, Germany) equipped with nitrogen 337 nm laser beam. Samples (0.8 μL) were mixed with 2,5-dihydroxybenzoic acid matrix (1.6 μL, 10 mg/mL in 30% acetonitrile and 0.1% TFA), applied to a MTP 384 ground steel target plate TF (Bruker Daltonics), and air-dried. Data were collected using Bruker’s flexControl software and the spectra were analyzed using Bruker’s flexAnalysis software.

## Results

### Phylogenetic and structural analysis of *Mt*LPMO9J

The MYCTH_79765 gene encodes a single AA9 domain of 229 residues with a theoretical molecular weight of 24.3 kDa (Figure AA in S1 File). SignalP 4.0 [45] predicted a 17-residue signal peptide, which was confirmed by experiment (see below). A structure based multiple sequence alignment (MSA) of the catalytic modules of *Mt*LPMO9J and 25 different LPMO9s, including several of known structure, revealed the conservation of the N-terminal His1, His83 and Tyr168, which compose the copper-binding motif (Fig 1B). Of the characterized LPMO9s, the closest homologs are *Nc*LPMO9C (63% sequence identity) and *Pa*LPMO9H (58% sequence identity), both of which have an additional CBM1 carbohydrate-binding module at the C-terminus. Phylogenetic analysis places *Mt*LPMO9J in a cluster together with C4-oxidizing LPMOs; this cluster is divided into two groups (Fig 1A). The group with *Mt*LPMO9J consists of LPMOs (*Nc*LPMO9C and *Pa*LPMO9H) that are active on cello-oligosaccharides; the other group contains *Mt*LPMO9C (MYCTH_100518) [13], which is not active on cello-oligosaccharides, and *Nc*LPMO9A (NCU02240) and *Nc*LPMO9D (NCU01050), LPMOs that accumulate C4-oxidized cellopentaose during their action on PASC [19] and, therefore, are likely not active on cello-oligosaccharides. Interestingly, two other cello-oligosaccharide-active LPMOs, *Ls*LPMO9A [35] and *Cv*LPMO9A [28], form a more distant cluster.

To assess putative structural differences between C4-oxidizing LPMO9s that are active and inactive on cello-oligosaccharides, a structural model of *Mt*LPMO9J was built and compared with the crystal structures of *Cv*LPMO9A (PDB: 5NLT) [28], *Nc*LPMO9A (PDB:5FOH), *Nc*LPMO9C (PDB:4D7U) [34], *Nc*LPMO9D (PDB:4EIR) [46] and *Ls*AA9A (PDB:5ACF) [28]. The MSA (Fig 1B) and structural comparisons (not shown) showed that sequence and structural diversity are concentrated in the so-called L2, L3, LS and LC regions [34] (Fig 1B). The surface of *Mt*LPMO9J contains several of the residues that are known to be involved in substrate binding, based on the structure of *Ls*AA9A in complex with cellohexaose [35] and NMR studies of *Nc*LPMO9C in complex with cellulose and xyloglucan [47] (Fig 1C and 1D). Residues involved in substrate binding show considerable but far from absolute conservation. The sequence alignment of Fig 1B does not reveal clear trends as to which substrate binding residues correlate with the ability to cleave soluble substrates. Residue 80 deserves attention because it has been suggested that the nature of this residue is correlated with oxidative regioselectivity [34, 48], although recent mutagenesis work suggests that this is not the case [49]. The residue is both close to the solvent exposed distal axial coordination position of the copper and close to the bound substrate [28, 35]. Notably, four of five LPMOs acting on soluble substrates have an alanine in this position, whereas LPMOs not active on soluble substrates have an aspartate (as does *Cv*LPMO9A that is active on cello-oligomers) (Fig 1B). Interestingly, *Mt*LPMO9J is the only enzyme in our analysis to present an extra Tyr residue (Y160) potentially involved in substrate binding (Fig 1B and 1D).

### Production and biophysical characterization of *Mt*LPMO9J

*Mt*LPMO9J was recombinantly produced in *A*. *nidulans* using the native signal peptide (residues 1–17), yielding approximately 12 mg of enzyme per L of culture medium. The enzyme was purified in two chromatographic steps to ca. 95% purity (Figure AA in S1 File). LC-MS analysis, discussed in more detail below, showed that the signal peptide of *Mt*LPMO9J was correctly processed, since in 98.6% of detected peptides containing the N-terminal histidine; this histidine was indeed residue number 1. LC-MS analysis further indicated that the N-terminal histidine was methylated in about 70% of the protein molecules (Table B in S1 File; more discussion below). An alternative construct using the glucoamylase signal peptide from the pEXPYR vector was also tested and resulted in the expression of inactive protein, suggesting incorrect processing of the signal peptide. The presence of Cu in the active site of purified *Mt*LPMO9J was confirmed by EPR spectroscopy (Figure AB in S1 File), which showed a spectrum characteristic of a mononuclear Cu center.

Next, we examined possible oxidative damage of *Mt*LPMO9J, which could have occurred during protein expression, for example as a result of auto-catalytic oxidation of the catalytic histidines recently described by Bissaro et al. [10]. In-depth LC-MS/MS analysis of trypsinated purified *Mt*LPMO9J showed oxidative damage to three of the enzyme’s four histidines, which are all close to the catalytic center. Based on peptide counts (Table B in S1 File), the N-terminal histidine was non-modified in 27% of the proteins, methylated in 70% and oxidized in 3%. The second catalytic histidine (His83) was not modified in 83% of the proteins, whereas it was oxidatively damaged in 17%. Concerning the two additional histidines close to the catalytic center, 10% of the proteins showed oxidative damage to His64, whereas damage to His157 could not be assessed due to lack of detected tryptic peptides.

**Fig 2:**
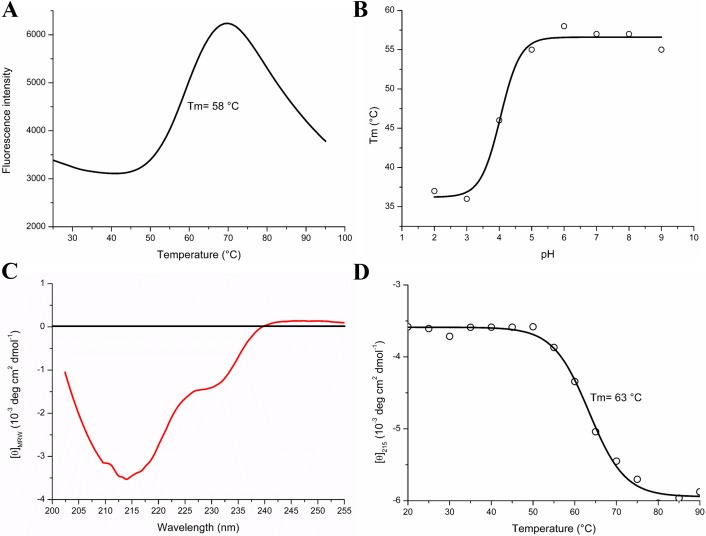
Thermal denaturation of *Mt*LPMO9J monitored by the ThermoFluor assay and CD spectroscopy. (A) Thermal shift curve of *Mt*LPMO9J at pH 6.0. The fluorescence was monitored and the peak temperature in Δϕ/Δt curve was used to extract the apparent melting temperature (T_m, app_). The protein concentration was 1.5 mg/mL and the heating rate was 1°C/min. (B) Thermal shift-based apparent melting temperatures obtained at different pHs. (C) Far-UV CD spectrum of *Mt*LPMO9J (red line). (D) Melting curve based on the CD signal at 215 nm, at pH 8.0; the protein concentration was 0.5 mg/mL and the heating rate was 1°C/min.

The structural stability of *Mt*LPMO9J as a function of pH and temperature was evaluated using a ThermoFluor assay and far-UV circular dichroism (CD). Protein denaturation curves measured using the reporter dye SYPRO Orange at different pHs showed structural stability at pHs above 5.0 with a melting temperature (T_m,app_) of 58°C (Fig 2A and 2B). Unfolding was irreversible and was also monitored, at pH 8.0, using the CD signal at 215 nm (Fig 2C and 2D), which yielded an apparent T_m,app_ of 63°C.

### Regiospecificity of *Mt*LPMO9J on cellulose

**Fig 3:**
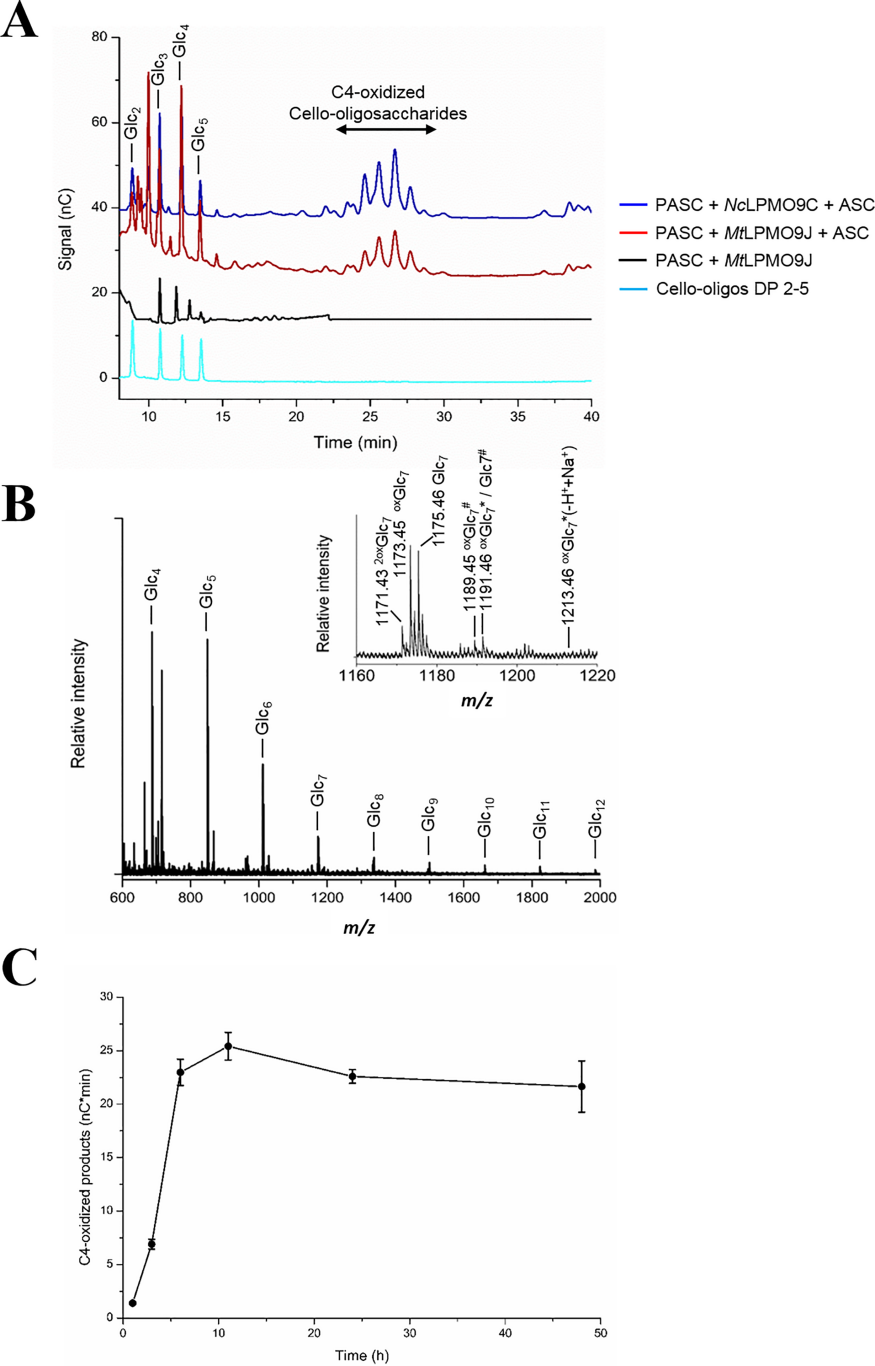
Product profile of *Mt*LPMO9J on PASC. (A) HPAEC-PAD chromatogram showing products released by *Mt*LPMO9J action on PASC. PASC (0.1%, w/v) was incubated with 5 μM *Mt*LPMO9J in 20 mM sodium acetate buffer (pH 6.0) containing 1 mM ascorbic acid (ASC) at 50°C for 16 h. Peaks were assigned based on native cello-oligosaccharide standards (black line) and the product profile of the C4-oxidizing LPMO *Nc*LPMO9C [24]. (B) MALDI-TOF/MS spectrum of cello-oligosaccharides released by *Mt*LPMO9J from PASC. The inset shows a close-up view of the Glc_7_ cluster. The masses of sodium or potassium adducts of native and oxidized species are labelled. Single or double oxidation is labelled with “ox” and “2ox”, respectively; hydration of the oxidized species is indicated by an asterisk; potassium adducts are labeled by hash; parentheses indicate uncertainty discussed in the main text. (C) Accumulation of C4-oxidized products over time during incubation of *Mt*LPMO9J with PASC. The amount of C4-oxidized products was calculated as sum of the peak areas for peaks corresponding to C4-oxidized products, eluting in the range of 22.5–30 min during HPAEC-PAD (see Figure D in S1 File for the chromatograms).

Incubation of *Mt*LPMO9J with phosphoric acid-swollen cellulose (PASC) resulted in the formation of native (i.e. non-oxidized) and oxidized cello-oligosaccharides in the presence of ascorbic acid as electron donor. HPAEC-PAD and MALDI-TOF/MS analyses of reaction mixtures with PASC showed the accumulation of cello-oligosaccharides with a degree of polymerization (DP) of 2 to 12 (Fig 3A and 3B). The HPAEC-PAD profile of *Mt*LPMO9J on PASC was similar to that of the C4-oxidizing *Nc*LPMO9C [24], showing only native and C4-oxidized cello-oligosaccharides (Fig 3A). MALDI-ToF/MS analysis confirmed C4-oxidation: the majority of the oxidized products were in the anhydrated 4-ketoaldose form (*m/z* 1173.45 for DP7) with minor amounts occurring in the hydrated gemdiol form (*m/z* 1191.46), while no double-Na^+^ adduct (*m/z* 1213.46), which is diagnostic of C1-oxidation [48], was detected (Fig 3B). The signal at *m/z* 1171.43 could, in principle, be an oligomer oxidized at both ends, but since there are no other, and more reliable, indications for C1-oxidation (i.e. a signal for a double sodium adduct), the *m/z* 1171.43 signal likely represents a degradation product. Notably, C4-oxidized cello-oligosaccharides are unstable, which also explains why one sees a relatively high amount of native products (on-column degradation of C4-oxidized products leads to generation of native products that are one residue shorter than the original oxidized product; see [50] for further discussion).

Following the release of C4-oxidized cello-oligosaccharides from PASC over time indicated that, under the conditions used, *Mt*LPMO9J remained active for about 6 hours, after which the activity declined and slow (although statistically not significant) degradation of the oxidized products became noticeable (Figs 3C and S4).

### Activity of *Mt*LPMO9J on cello-oligosaccharides

In addition to polymeric cellulose, *Mt*LPMO9J was also active on water-soluble cello-oligosaccharides. The enzyme cleaved cellohexaose (Glc_6_) to produce native and C4-oxidized trimers (Glc_3_ and ^ox^Glc_3_) or a native tetramer and a C4-oxidized dimer (Glc_4_ and ^ox^Glc_2_) (Figure B in S1 File; subsites derived from this cleavage pattern are indicated in Fig 1C), while cellopentaose (Glc_5_) was converted to a native cellotriose and a C4-oxidized dimer (Glc_3_ and ^ox^Glc_2_). Activity on cellotetraose (Glc_4_) and cellotriose (Glc_3_) was not detected (Figure B in S1 File).

### Substrate specificity of *Mt*LPMO9J

**Fig 4:**
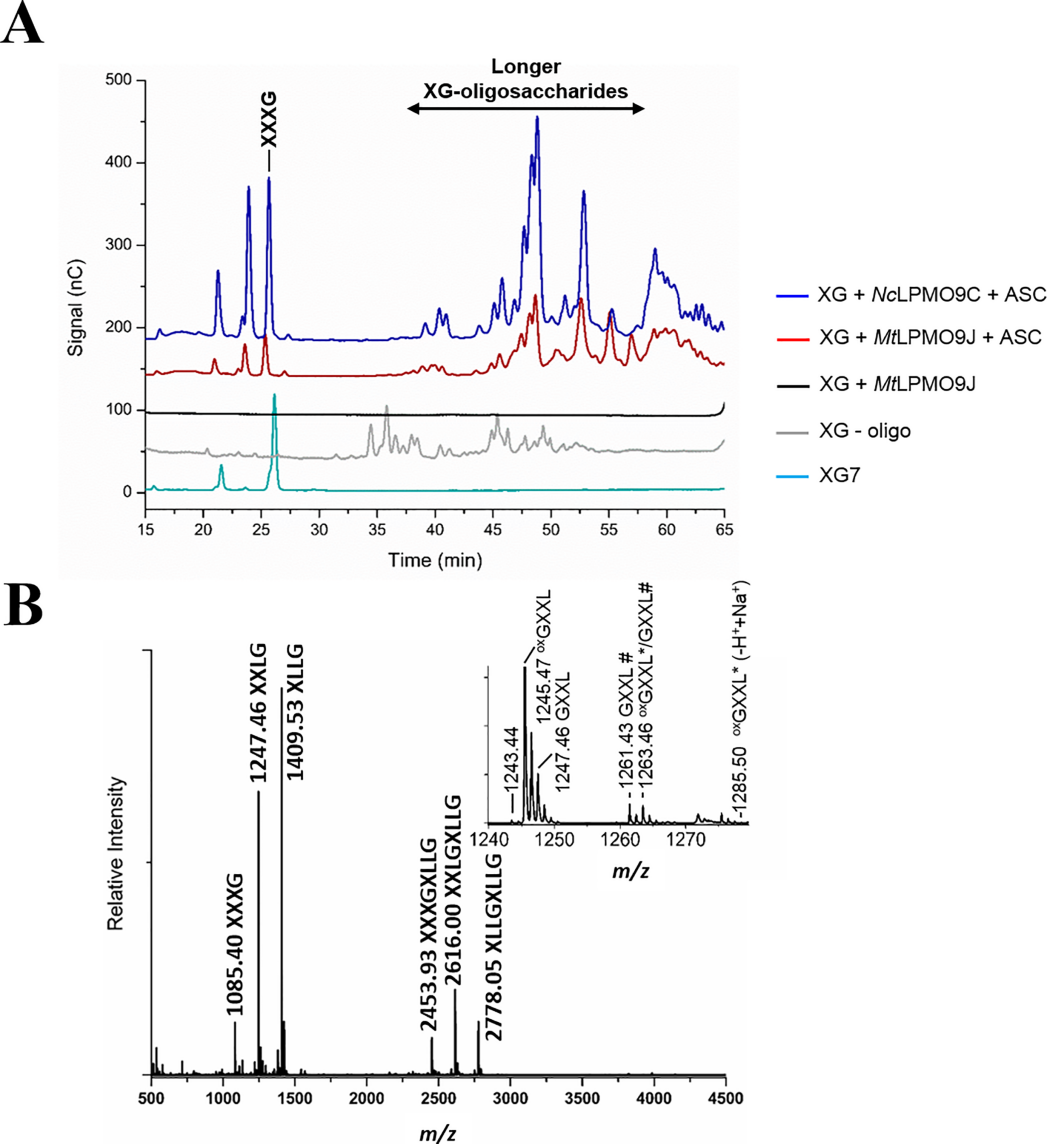
Products generated by *Mt*LPMO9J from tamarind xyloglucan. (A) HPAEC-PAD chromatogram showing the products released after *Mt*LPMO9J action on tamarind xyloglucan (brown line). Tamarind xyloglucan (0.1%, w/v) was incubated with 5 μM *Mt*LPMO9J in 20 mM sodium acetate buffer (pH 6.0) containing 1 mM ascorbic acid (ASC) at 50°C for 16 h. *Nc*LPMO9C was used as positive control (blue line); a commercially available mixture of xyloglucan-oligosaccharides (DP 14–27, grey line) and a xyloglucan heptamer (XG7, turquoise line) were used as standards. (B) MALDI-TOF/MS spectrum of xyloglucan-oligosaccharides released by *Mt*LPMO9J from tamarind xyloglucan. In the main panel, Na^+^ adducts of native products are labelled, although oxidized products dominate the clusters. The inset shows a close-up view of the Hex_5_Pen_3_ cluster; the masses of native and oxidized species are labelled. Oxidation is labelled with “ox”; hydration of the oxidized species is labelled with an asterisk; all adducts are sodium adducts except for those labeled with hash, which are potassium adducts. The xyloglucan oligosaccharides have the following nomenclature: G, a non-substituted glucose unit; X, a G unit *O*-6-substituted with a xylose unit; L, an X unit where xylose *O*-2-substituted with a galactose unit.

As some of the closest homologs of *Mt*LPMO9J are active on hemicelluloses, the activity of *Mt*LPMO9J was assayed against tamarind xyloglucan, konjac glucomannan, barley β-glucan and sugar beet arabinan. Of these substrates, only tamarind xyloglucan was cleaved (Fig 4 and Figure C in S1 File). The product profile (HPAEC-PAD and MALDI-TOF/MS) for *Mt*LPMO9J was similar to that of *Nc*LPMO9C [23]. MALDI-ToF/MS data (Fig 4B) showed a clustered product profile, indicating a preference for chain cleavage at non-substituted glucose units. This implies that products contain a multitude of three pentoses, as was indeed observed. (Of note, while the predominant repeating units of tamarind xyloglucan, XXXG, XXLG and XLLG, contain three xyloses, and occasional arabinose unit may occur). A close-up of the Hex_5_Pen_3_ cluster shows that the predominant species was the anhydrated form of the oxidized product (*m/z* 1245.47), while the hydrated form (*m/z* 1263.46) was also detected. The lack of the *m/z* 1285.50 signal (corresponding to the Na^+^-adduct of the Na^+^-salt of the C1-oxidized species) indicates the absence of C1-oxidation. Of note, despite major purification efforts, the purified enzyme displayed a background xylanase activity originating from the expression host, which precluded assessment of the activity of *Mt*LPMO9J on xylan and xylo-oligosaccharides.

## Discussion

Thermophilic fungi, such as *Myceliophthora thermophila*, are a promising source of thermostable enzymes able to hydrolytically and oxidatively degrade plant cell wall components. Application of thermostable enzymes, including LPMOs, for biomass conversion could enhance saccharification rates and decrease risk of microbial contamination. Here we report the characterization of an LPMO9 from *M*. *thermophila*, *Mt*LPMO9J (MYCTH_79765)[26], obtained by heterologous expression in *A*. *nidulans*. This enzyme has been previously identified among 11 *Mt*LPMO9s, including the five previously characterized *Mt*LPMO9s [13, 19, 27], which are secreted by *M*. *thermophila* when grown on alfalfa and barley straw [26] (Table A in S1 File).

To date, just a few LPMOs have been reported to cleave cello-oligosaccharides, including *Nc*LPMO9C from *N*. *crassa* [24], *Pa*LPMO9H from *P*. *anserina* [51], *Ls*AA9A from *L*. *similis* [35] and *Cv*AA9A from *C*. *virescens* [28], *Nc*LPMO9C and *Pa*LPMO9H being the closest characterized homologs of *Mt*LPMO9J (Fig 1A). *Mt*LPMO9J showed the same cleavage pattern on cellohexaose and cellopentaose as *Nc*LPMO9C [24] and *Pa*LPMO9H [51].

Regarding hemicellulose oxidation, *Mt*LPMO9J showed activity on xyloglucan, cleaving the substrate adjacent to unsubstituted glucosyl units. We could not detect LPMO activity on xylan, arabinan, glucomannan or β-glucan. Interestingly, the closely related *Nc*LPMO9C is active on glucomannan and β-glucan. There is no obvious explanation for these observed differences but we note considerable sequence variation among LPMOs, for example in the highly variable LC region.

The genomes of fungi tend to encode large numbers of LPMOs [25] and it remains to be seen to what extent the encoded LPMOs are functionally redundant or complementary. It is to be expected that additional functionalities and subtle differences in known functionalities await discovery. The current study adds a sixth enzyme to the list of LPMO9s from *M*. *thermophila* that have been characterized. Although not all these LPMOs have been characterized to similar depths, it is of interest to compare their known properties, as we do in Table A in S1 File. Already now, with only a fraction of *Mt*LPMO9s having been characterized, considerable functional diversity has been disclosed. More functional and structural studies are needed to uncover the total LPMO-catalyzed oxidizing power of *M*. *thermophila*. Of note, a general consensus on the nomenclature of the newly characterized *Mt*LPMO9s seems to be lacking.

Several questions remain as to the mechanism of LPMOs, and the question whether O_2_ or H_2_O_2_, as proposed by Bissaro *et al*. in 2016 [52], is the biologically relevant co-substrate is still debated. While this report was being completed, two publications appeared describing detailed kinetic analyses of the action of *Mt*LPMO9J on cellohexaose and addressing the possible roles of O_2_ or H_2_O_2_ ([53, 54]; note that the enzyme, accession codes MYCTH_79765, or, in UniProt, G2Q7A5, is referred to as *Mt*LPMO9E in these publications). In one of these reports [54], Hangasky *et al*. show that LPMOs indeed can use H_2_O_2_ quite efficiently, supporting the findings by Bissaro et al [10, 52], but conclude from the sum of their experiments that O_2_ is the natural co-substrate, in contrast with the conclusions drawn by Bissaro *et al*. As we did not quantify LPMO activity on the cello-oligosaccharides, a direct comparison of our results with the reaction rates for cellohexaose degradation reported by Hangasky *et al*. [53] is not possible. Both studies demonstrate clear activity on cellohexaose and cellopentaose, whereas, in contrast to the data presented here, Hangasky *et al*. also detected a minor activity on cellotetraose.

Recent work on LPMOs has revealed that these enzymes are prone to oxidative self-inactivation, e.g. under conditions where they are reduced in the absence of substrate [52, 54]. Indeed, non-linear progress curves caused by enzyme inactivation are a commonly observed phenomenon in studies of LPMOs (e.g. [10, 18], Fig 3C). Importantly, we show here that heterologously expressed, purified *Mt*LPMO9J, which had never been exposed to reaction conditions, such as an added reductant, carried oxidative damage in the catalytic center (Table B in S1 File). It is conceivable that the LPMO experiences damage-inducing conditions (i.e. reduction in the absence of substrate and the presence of oxygen or H_2_O_2_) during the expression and purification protocols. From a practical point of view, our finding of enzyme oxidation during production is potentially of major importance. Oxidatively damaged LPMOs will not be recognized as such when using common quality control methods for proteins like SDS-PAGE or gel filtration. Nevertheless, such LPMOs will be impaired in copper binding and catalytic performance.

## Supporting information

S1 File Supporting information for *Mt*LPMO9J. (PDF)
