# Supplementary material for: Functional characterization of a lytic polysaccharide monooxygenase from the thermophilic fungus Myceliophthora thermophila
Source: PLoS One. 2018 Aug 20;13(8):e0202148. doi: 10.1371/journal.pone.0202148 (PMC6101365; doi:10.1371/journal.pone.0202148)
Supplement: S1 File — (PDF) [file pone.0202148.s001.pdf]

S1. Supporting information for *Mt*LPMO9J

Functional characterization of a lytic polysaccharide monooxygenase from the thermophilic fungus *Myceliophthora thermophila*

Marco AS Kadowaki[1,¶], Anikó Várnai[2, ¶], John-Kristian Jameson[2], Ana E T Leite[1], Antonio J. Costa-Filho[3], Patricia S. Kumagai[1], Rolf A Prade[4], Igor Polikarpov[1,*], Vincent GH Eijsink[2,*]

[1] Department of Physics and Interdisciplinary Science, São Carlos Institute of Physics, University of São Paulo, São Carlos, São Paulo, Brazil

[2]Faculty of Chemistry, Biotechnology and Food Science, Norwegian University of Life Sciences (NMBU), Ås, Norway

[3]–Department of Physics, Faculdade de Filosofia, Ciências e Letras de Ribeirão Preto, University of São Paulo, Ribeirão Preto, São Paulo, Brazil

[4]Departments of Biochemistry & Molecular Biology and Microbiology & Molecular Genetics, Oklahoma State University, Stillwater, OK, United States

¶These authors contributed equally to the work.

[*]Corresponding authors:

E-mails: vincent.eijsink@nmbu.no; ipolikarpov@ifsc.usp.br.

Table A. Overview of LPMOs encoded in the genome of *Myceliophthora thermophyla*. The locus tag refers to the *Thermothelomyces thermophila* ATCC 42464 (Spoth2 in JGI) genome. The databases (JGI genome and CAZy protein databases) where the LPMOs are listed are given under "source"; the nomenclature was adapted from Berka *et al* [1]. The protein modules were identified with dbCAN; secretion on alfalfa and barley straw is reported according to Berka *et al* [1]. The regioselectivity on cellulose, substrate specificity (phosphoric acid swollen cellulose, PASC; cello-oligosaccharides, COS; xyloglucan, XG; β-glucan, BG; xylan associated to cellulose, X/C) and expression host of the previously characterized *Mt*LPMOs and the hereby characterized *Mt*LPMO9J are indicated.

No.	Locus tag	Source	Annotation by Berka *et al* [1]	Protein modules	Secreted on alfalfa and barley straw [1][g]	Regioselectivity	Substrate reported	Expression host	Reported name and reference
1	MYCTH_46583	JGI; CAZy; [1]	LPMO9H	AA9, CBM1	Yes	C1/C4	PASC	*P. pastoris*	*Mt*LPMO9I [2]
2	MYCTH_47093[b]	JGI; CAZy; [1]	LPMO9Q	AA9	Not reported				
3	MYCTH_55803	JGI; CAZy; [1]	LPMO9F	AA9	No				
4	MYCTH_79765	JGI; CAZy; [1]	LPMO9J	AA9	Yes	C4	PASC, COS, XG	*A. nidulans* A773<br>*N. crassa*	*Mt*LPMO9J, this study<br>*Mt*PMO9E [3]
5	MYCTH_80312	JGI; CAZy; [1]	LPMO9B	AA9, CBM1	Yes	C1	PASC	*M. thermophyla* C1	*Mt*LPMO9B [4]
6	MYCTH_85556	JGI; CAZy; [1]	LPMO9E	AA9	Yes	C1/C4	PASC, XG, BG, X/C	*M. thermophyla* C1	*Mt*LPMO9A [5]
7	MYCTH_92668	JGI; CAZy; [1]	LPMO9D	AA9	Yes	C1	PASC	*N. crassa*<br>*M. thermophyla* C1	MYCTH_92668 [6]<br>*Mt*LPMO9D [7]
8	MYCTH_96032	JGI; CAZy; [1]	LPMO9R	AA9	No				
9[a]	MYCTH_98122<br>MYCTH_2112799	JGI<br>CAZy; [1]	–[f]<br>LPMO9P	AA9<br>AA9	Not reported<br>Yes				
10	MYCTH_100518	JGI; CAZy; [1]	LPMO9W	AA9	Yes	C4	PASC, XG, BG	*M. thermophyla* C1	*Mt*LPMO9C [4]
11	MYCTH_103537	JGI; CAZy; [1]	LPMO9L	AA9	Yes				

Table A. Overview of LPMOs encoded in the genome of *Myceliophthora thermophyla*. Continued.

No.	Locus tag	Source	Annotation by Berka *et al* [1]	Protein modules	Secreted on alfalfa and barley straw [1]	Regio-selectivity	Substrate reported	Expression host	Reported name and reference
12	MYCTH_110651	JGI; CAZy; [1]	LPMO9G	AA9, CBM1	Yes				
13	MYCTH_111088	JGI; CAZy; [1]	LPMO9I	AA9, CBM1	Yes				
14	MYCTH_112089	JGI; CAZy; [1]	LPMO9A	AA9	Yes	C1	PASC	*N. crassa*	MYCTH_112089 [6]
15[a]	MYCTH_2044732[c]<br>MYCTH_2060403	JGI<br>CAZy; [1]	–[f]<br>LPMO9O	AA9<br>AA9	Not reported<br>Not reported				
16[a]	MYCTH_2053130<br>MYCTH_2054500	JGI; [1]<br>CAZy; [1]	LPMO9<br>LPMO9S	AA9<br>AA9	Not reported<br>No				
17[a]	MYCTH_2063480[d]<br>MYCTH_116175[d]	JGI; [1]<br>CAZy	LPMO9M<br>–[f]	AA9<br>AA9	Not reported<br>Not reported				
18[a]	MYCTH_2130287<br>MYCTH_2035750[e]	JGI<br>[1]	–[f]<br>LPMO9K	AA9, CBM1<br>AA9, CBM1	No<br>Not reported				
19	MYCTH_2298502	JGI; CAZy; [1]	LPMO9V	AA9	No				
20	MYCTH_2299721	JGI; CAZy; [1]	LPMO9Y	AA9	No				
21	MYCTH_2301632	JGI; CAZy; [1]	LPMO9X	AA9	No				
22	MYCTH_2306673	JGI; CAZy; [1]	LPMO9N	AA9	No				
23	MYCTH_2311323	JGI; CAZy; [1]	LPMO9T	AA9	No				

[a] LPMO genes with alternative splicing are listed under the same number.

[b] Fragment only.

[c] Stop codon missing.

[d] Missing/wrong signal peptide and missing His1.

[e] Stop codon missing; transcript and translation to protein do not match in the JGI database.

[f] Not listed in Berka *et al* [1].

[g] Cultivation conditions: submerged culture with 2% (w/v) alfalfa or barley straw (ground to 0.5 cm length) as carbon source, incubated at 34°C or 45°C with shaking at 150 rpm for 21 h.

Table B. Oxidation of histidines near the catalytic center in purified *Mt*LPMO9J. Peptides were analyzed by LC-MS/MS and their identity and modifications were identified with the Mascot server using error tolerant searches. The table shows the total number of detected peptides containing a histidine and the occurrence of the indicated modifications on these peptides (in absolute numbers and % of total). The main modification of the histidines are methylation (*m/z*= +14), and oxidation (*m/z*= +16), where the latter may lead to subsequent ring opening to form aspartate (*m/z*= -22) or asparagine (*m/z*= -23) [8]. Tryptic peptides containing the fourth histidine in *Mt*LPMO9J, His157, were not detected.

	His1	His64	His83
**Unmodified**	83 (27%)	196 (90%)	81 (83%)
**Oxidized**	-	21 (10%)	6 (6.2%)
**Methylated**	220 (70%)	-	-
**Aspartate**	7 (2.4%)	-	9 (9.3%)
**Asparagine**	2 (0.6%)	-	1 (1%)
**Total peptides**	312	217	97

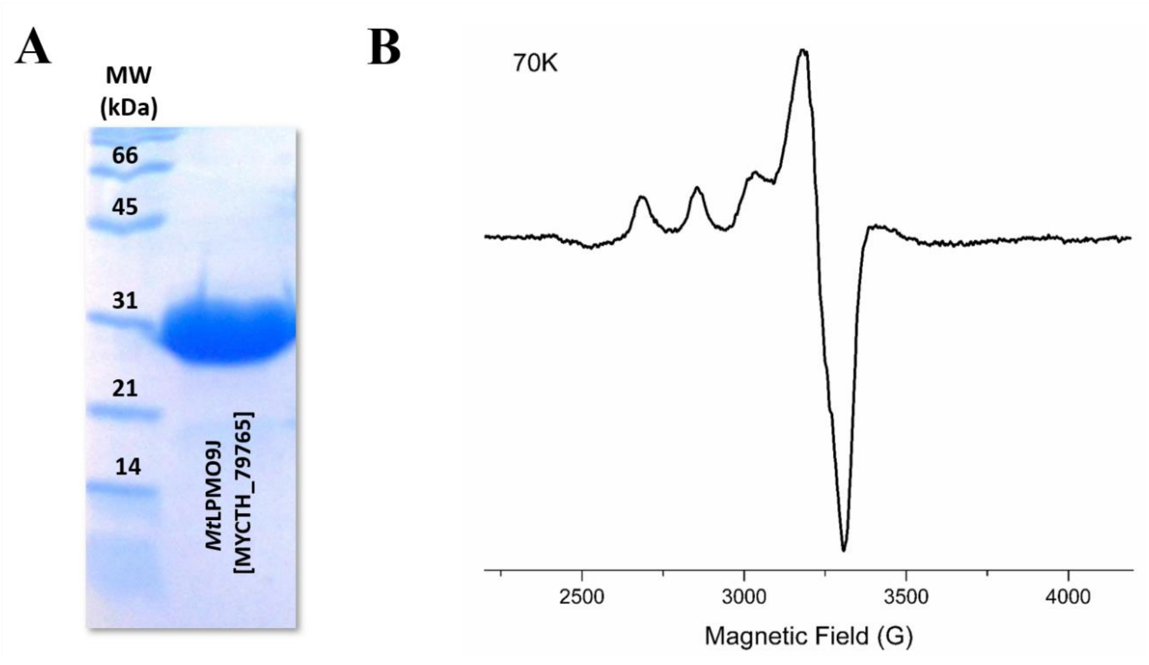

Fig A. A) SDS-PAGE and B) electron paramagnetic resonance (EPR) analysis of purified *Mt*LPMO9J.

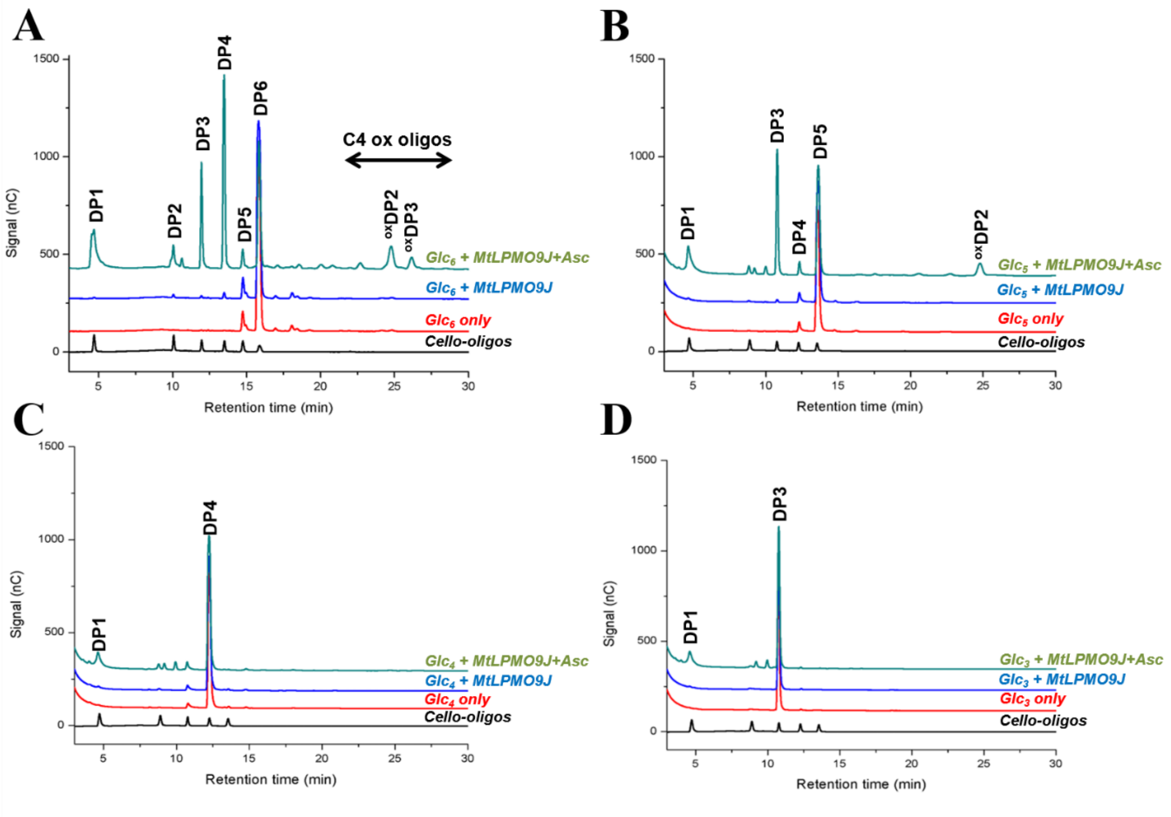

Fig B. HPAEC-PAD profiles of products generated by *Mt*LPMO9J from cello-oligosaccharides. In the reaction, 0.5 mM (A) cellohexaose (Glc$_6$), (B) cellopentaose (Glc$_5$), (C) cellotetraose (Glc$_4$) or (D) cellotriose (Glc$_3$) was treated with 5 µM *Mt*LPMO9J in 20 mM sodium acetate buffer (pH 6.0) containing 1 mM ascorbic acid as electron donor. Controls were included without ascorbic acid (blue lines) and with substrate only (red lines); native cello-oligosaccharides with DP1-6 or DP 1-5 (black line) were used as standard. Note that the C4-oxidized products are unstable and will be converted to native products that are one sugar shorter than the original oxidized product [50]; this explains the appearance of a monomer peak in panels A and B. The apparent monomer peak in panels C and D represents a background signal.

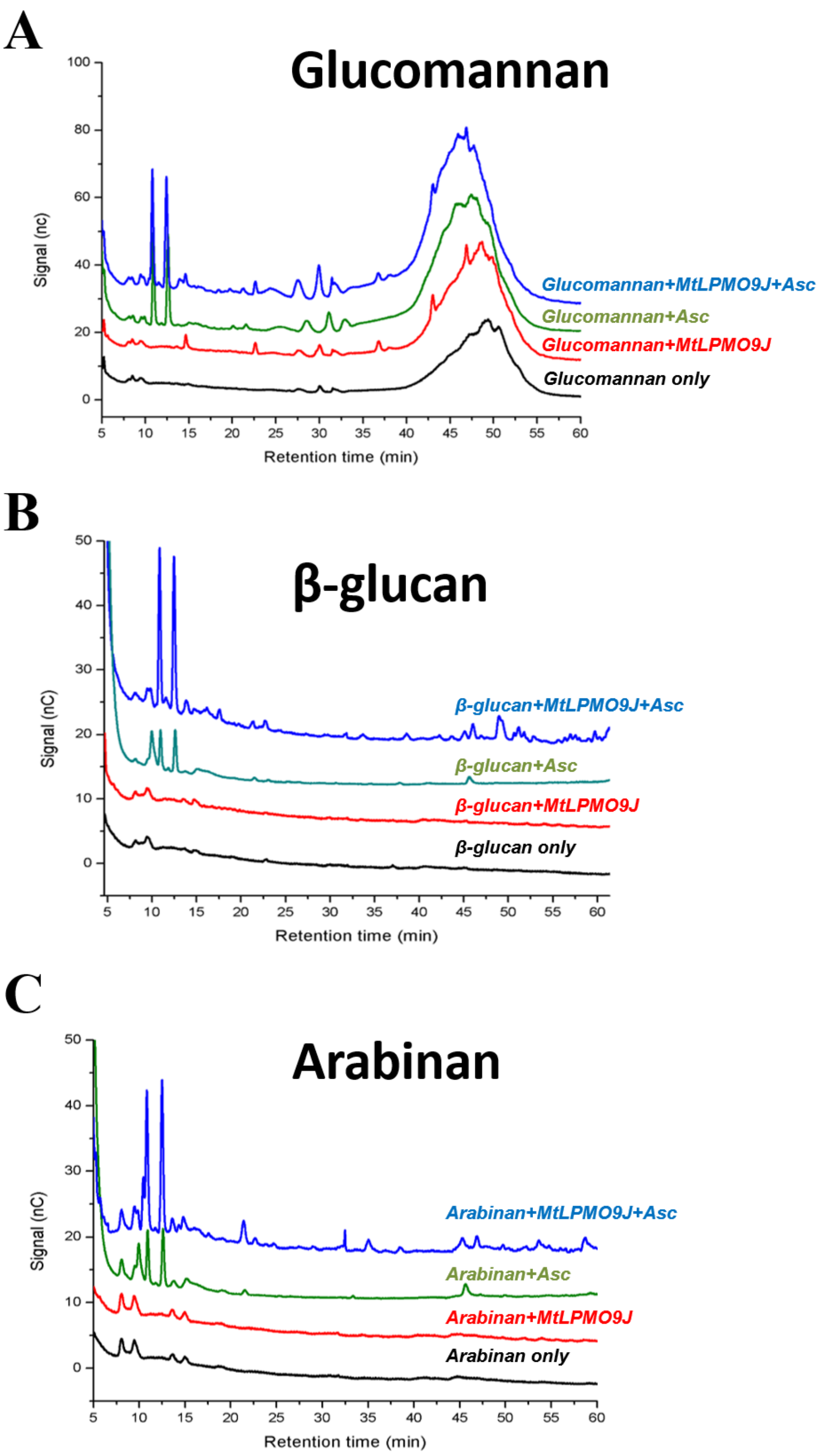

Fig C. HPAEC-PAD profiles of products generated by *Mt*LPMO9J from hemicellulosic substrates. In the reaction, konjac glucomannan (A), barley β-glucan (B) or sugar beet arabinan (C) at 0.1% (w/v) was incubated with 5 µM *Mt*LPMO9J in 20 mM sodium acetate buffer (pH 6.0) containing 1 mM ascorbic acid (ASC) at 50 °C for 16 h. Controls were included with ascorbic acid only (green lines), without ascorbic acid (red lines) and substrate only (black lines).

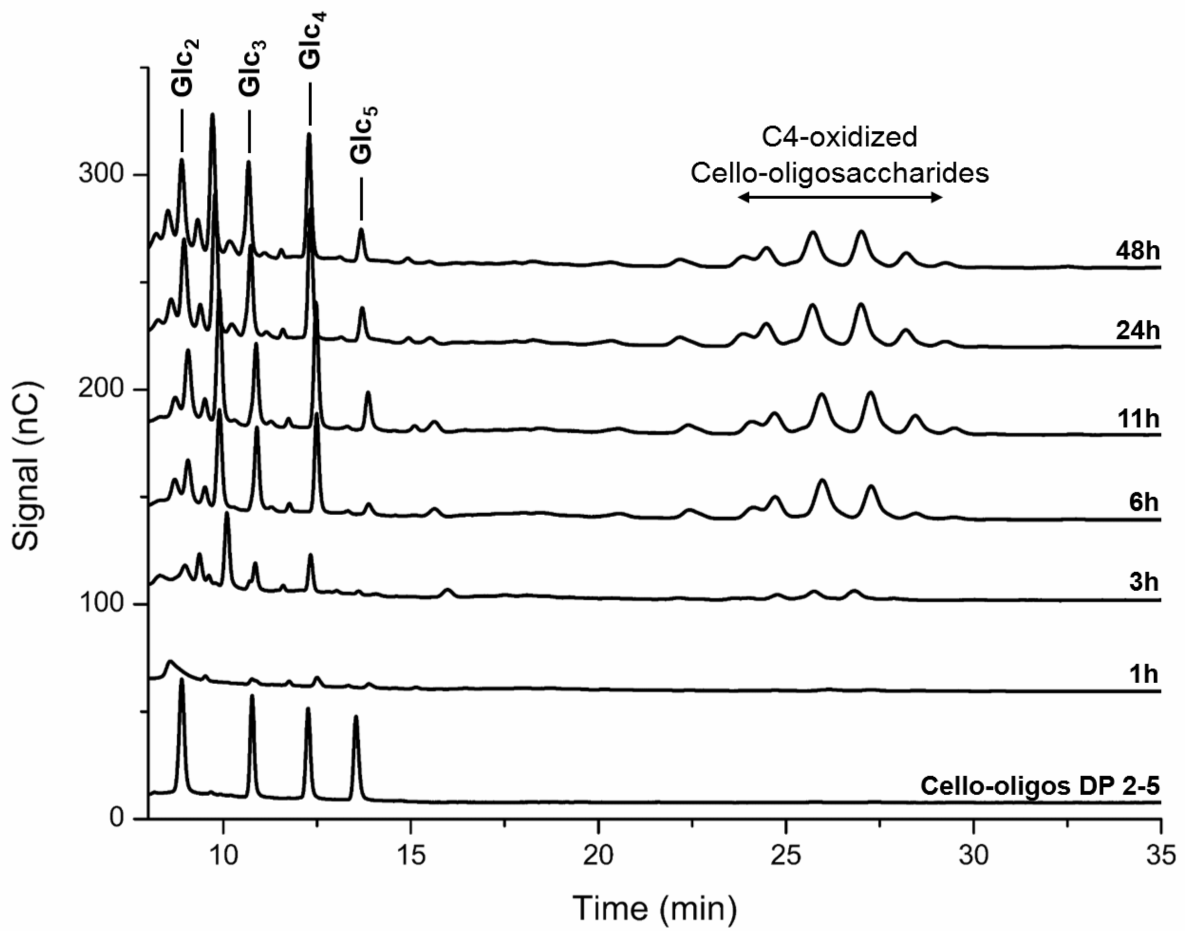

Fig D. HPAEC-PAD profiles of products generated by *Mt*LPMO9J from PASC over time. Enzymatic assays were performed at 50 °C in 20 mM sodium acetate buffer (pH 6.0); the reactions contained 0.1% PASC, 5 µM *Mt*LPMO9J and 1 mM ascorbic acid. Native cello-oligosaccharides with DP 2-5 were used as standard. Note that native products emerge in part as a result of on-column degradation of C4-oxidized products, as outlined in [9].

References

1. Berka, R.M., et al., *Comparative genomic analysis of the thermophilic biomass-degrading fungi Myceliophthora thermophila and Thielavia terrestris.* Nat Biotechnol, 2011. **29**(10): p. 922-7.
2. Karnaouri, A., et al., *Recombinant expression of thermostable processive MtEG5 endoglucanase and its synergism with MtLPMO from Myceliophthora thermophila during the hydrolysis of lignocellulosic substrates.* Biotechnol Biofuels, 2017. **10**: p. 126.
3. Hangasky, J. and M.A. Marletta, *A random-sequential kinetic mechanism for polysaccharide monooxygenases.* Biochemistry, 2018: p. 1-30.
4. Frommhagen, M., et al., *Lytic polysaccharide monooxygenases from Myceliophthora thermophila C1 differ in substrate preference and reducing agent specificity.* Biotechnol Biofuels, 2016. **9**(1): p. 1-17.
5. Frommhagen, M., et al., *Discovery of the combined oxidative cleavage of plant xylan and cellulose by a new fungal polysaccharide monooxygenase.* Biotechnol Biofuels, 2015. **8**: p. 101.
6. Vu, V.V., et al., *Determinants of regioselective hydroxylation in the fungal polysaccharide monooxygenases.* J Am Chem Soc, 2014. **136**(2): p. 562-5.
7. Frommhagen, M., et al., *Quantification of the catalytic performance of C1-cellulose-specific lytic polysaccharide monooxygenases.* Appl Microbiol Biotechnol, 2018. **102**(3): p. 1281-1295.
8. Uchida, K. and S. Kawakishi, *Ascorbate-mediated specific oxidation of the imidazole ring in a histidine derivative.* Bioorganic Chemistry, 1989. **17**(3): p. 330-343.
9. Westereng, B., et al., *Simultaneous analysis of C1 and C4 oxidized oligosaccharides, the products of lytic polysaccharide monooxygenases acting on cellulose.* J Chromatogr A, 2016. **1445**: p. 46-54.
